# Individual, Maternal, Household, and Community Level Variability in Determining Inequalities in Childhood Anaemia within Ethiopia: Four-Level Multilevel Analysis Approach

**DOI:** 10.3390/children9091415

**Published:** 2022-09-19

**Authors:** Kenenisa Abdisa Kuse, Demie Seyoum Jima, Teshita Uke Chikako, John Elvis Hagan, Abdul-Aziz Seidu, Richard Gyan Aboagye, Bright Opoku Ahinkorah

**Affiliations:** 1 Department of Statistics, Bule Hora University, Bule Hora P.O.Box 144, Ethiopia; 2 Wondo Genet College of Forestry and Natural Resource, Hawassa University, Hawassa P.O. Box 05, Ethiopia; 3 Department of Health, Physical Education, and Recreation, University of Cape Coast, Cape Coast PMB TF0494, Ghana; 4 Neurocognition and Action-Biomechanics-Research Group, Faculty of Psychology and Sport Sciences, Bielefeld University, Postfach 10 01 31, 33501 Bielefeld, Germany; 5 College of Public Health, Medical and Veterinary Sciences, James Cook University, Townsville, QLD 4811, Australia; 6 Centre for Gender, Takoradi Technical University, Takoradi P.O. Box 256, Ghana; 7 Department of Family and Community Health, Fred N. Binka School of Public Health, University of Health and Allied Sciences, Ho PMB 31, Ghana; 8 School of Public Health, Faculty of Health, University of Technology Sydney, Ultimo, NSW 2007, Australia

**Keywords:** anaemia, childhood, Ethiopia, multilevel logistic regression

## Abstract

**Background:** Childhood anaemia is a major public health issue necessitating rapid attention due to its debilitating consequences on the child, family, and society. Previous studies have assessed the prevalence and contributing factors to childhood anaemia in many developing countries. Yet, little is known about the factors that contribute to childhood anaemia in Ethiopia. The study examined the factors associated with inequalities of childhood anaemia in Ethiopia. **Methods:** Data for the study were extracted from the 2016 Demographic and Health Survey of Ethiopia. A total of 7960 children were considered in the final study. Bivariate and multilevel ordinal logistic regression analyses were used to estimate determinants of inequalities of childhood anaemia status. **Results:** Overall, the prevalence of mild, moderate, and severe anaemia among the children were 24.5%, 28.4%, and 2.2%, respectively. The child’s age (in months), sex of the child, preceding birth interval (in months), mother’s educational level, antenatal care visit, wealth index of mothers, source of drinking water, type of toilet facility, place of residence, and region were significantly associated with childhood anaemia. The multilevel random coefficient model found that there is a variation of childhood anaemia among women (intra-cluster correlation [ICC] = 15.06%), households (ICC = 15.6%), and communities (ICC = 14.22%) in Ethiopia. **Conclusions:** This study showed that anaemia is common among Ethiopian children. Factors found to be associated with childhood anaemia were the sociodemographic characteristics of the child and their mothers. We recommend that existing programs and interventions to prevent and reduce childhood anaemia be strengthened. Moreover, a targeted intervention includes deworming, intensified year-round behavior change communication campaigns and testing using digital methods, and point-of-care treatment.

## 1. Introduction

Anaemia, defined as a decrease in the concentration of circulating red blood cells or in the hemoglobin concentration and a concomitant impaired capacity to transport oxygen [1], is a major public health issue necessitating rapid attention due to the debilitating consequences it has on the individual, family, and their societies [2]. Among children under five, anemia is classified into mild (haemoglobin [Hb] =10.0–10.9 g/dL), moderate (Hb = 7.0–9.9 g/dL), severe (Hb < 7.0 g/dL), and normal (Hb ≥ 11.0 g/dL) Hb level concentration for children aged 6 to 59 months [2]. Signs and symptoms may include fatigue, skin pallor, shortness of breath, light-headedness, dizziness, or a fast heartbeat [3].

Anaemia is prevalent among pregnant women and children under five years of age, especially during their first two years of life. More than 1.62 billion people are anaemic worldwide, and approximately two-thirds of preschool children in Africa and South East Asia are anaemic [4]. The World Health Organization (WHO) listed iron deficiency, a major cause of anemia, as one of the top 10 risk factors in developing countries for “lost years of healthy life” [5,6]. Anaemia affects around 62.3% of sub-Saharan African children under five years of age [7]. Among preschool children in sub-Saharan Africa, anaemia is prevalent in 42% and 91% of children in Swaziland and Burkina Faso respectively [8].

Developing countries, particularly those in Africa and South-East Asia, account for over 89% of the global anaemia burden [9]. Aside from being disproportionately prevalent in these regions, anaemia prevalence varies by age group. Children under the age of five represent over 47% of the total burden [10], with Ethiopia having one of the highest rates [11].

Anaemia is a serious global public health problem affecting young children [4]. According to the WHO criteria, anaemia is a severe public health problem in Ethiopia, and 56.9% of Ethiopian under-five children are anemic, based on the Ethiopian Demographic and Health Survey (EDHS) [12]. This is a little lower than the average anaemia prevalence of 59.9% in under-five children from 27 Sub-Saharan African countries [13].

Low- and middle-income countries are more affected by the consequences of anaemia. For instance, a significant amount of human capital and economic resources are lost due to the premature morbidity and mortality of pregnant women and under-five children [14]. Anaemia impairs cognitive function during the early developmental stages of childhood, as previously reported in the literature [15], and affected children often suffer from other complications of iron and vitamin B_12_ deficiencies [15,16,17]. Long-term consequences include poor psychomotor skills, poor academic performance, and infection susceptibility [18,19]. Global food insecurity must therefore be addressed urgently as poor nutrition is the most common cause of anaemia [15,16,17].

Several interventions have been implemented in Ethiopia to address the high prevalence of anaemia among children, according to the EDHS 2011 report [20]. In the same report, more than four out of ten (44%) children in Ethiopia were anaemic. Thus, the prevalence of mild, moderate, and severe anaemia were 21%, 20%, and 3%, respectively. The prevalence of anaemia is highest in children aged 9–11 months (73%), and it decreases steadily from 12 to 59 months [20]. Children in rural areas have anaemia at a rate of 45 percent compared to children in urban areas, with a prevalence rate of 35 percent. Regional differences in anaemia in children range from 33% in Addis Ababa to 75% in Afar [20]. The national estimate of anaemia prevalence decreased by 19%, from 54% in 2005 to 44% in 2011 [20]. Evidence from the 2016 EDHS reports shows that more than half of children under five, around(57%), suffer from some degree of anaemia, 25% are mildly anaemic, 29% are moderately anaemic, and 3% are severely anaemic [12]. This increment of under-five child anaemic problems from year to year has received little research attention.

Studies have shown that socioeconomic, demographic, and environmental factors contribute to childhood anaemia [21,22,23,24]. Anaemia among children in Ethiopia has been associated with multiple risk factors in several studies [21,22,24,25]. However, these studies did not classify the severity of anaemia and its determinants. Therefore, this study examined socioeconomic, demographic, household, and environmental factors associated with inequality in childhood anaemia in Ethiopia.

## 2. Methodology

### 2.1. Data Source and Study Design

We performed a cross-sectional analysis of data from the EDHS. The survey was conducted from 18 January 2016, to 27 June 2016, based on a nationally representative sample that provides estimates at the national and regional levels for both urban and rural areas [8]. The EDHS utilized a descriptive cross-section design, relying on structured questionnaires to collect data from the respondents. EDHS employed a two-stage cluster sampling technique to recruit respondents for the survey. The detailed sampling procedure adopted for the EDHS can be found in the literature [12]. The dataset used is freely available to download at https://dhsprogram.com/data/dataset/Ethiopia_StandardDHS_2016.cfm?flag=1 (accessed on 8 January 2022).

### 2.2. Study Procedure

Blood specimens for testing anaemia were collected from all children aged 6–59 months for whom consent was obtained from their parents or other adults responsible for them. Blood samples were drawn from a drop of blood taken from a finger prick (or a heel prick in the case of children) and collected in a micro cuvette. Hemoglobin analysis was conducted on-site using a battery-operated portable HemoCue analyzer. The results were provided verbally and in writing. The parents or responsible adults of children whose Hb was below 7 g/dL were instructed to take the child to a health facility for follow-up care [12].

### 2.3. Study Variables

#### 2.3.1. Dependent Variable

The dependent variable was the childhood anaemic status, measured as the altitude-adjusted Hb concentration (in g/L), and the severity of anaemia was categorized as [2].

$$\mathrm{Anaemic}\;\mathrm{status}\{\begin{array}{c} 1=mild\left(100-109\frac{g}{L}\right),\\ 2=moderate\;(70\;-\;99g/L)\\ 3=severe\;(<70g/L) \end{array}$$

#### 2.3.2. Independent Variables

Independent variables considered in this study were grouped by socioeconomic, demographic, household, and environmental factors.

#### 2.3.3. Individual-Level Factors

Child’s age (in months) (0 = “<6”, 1 = “6–11”, 2 = “12–23”, 3 = “24–37”, 4 = “38–47”, 5 = “48–59”), Sex of child (0 = “Male”, 1 = “Female”), Preceding birth interval (in months) (0 = “<24”, 1 = “≥24”), Size of child at birth (0 = “Very Large”, 1 = “larger than Average”, 2 = “Medium”, 3 = “Smaller than Average”, 4 = “very Small”).

#### 2.3.4. Maternal-Level Factors

Women’s Age (0 = “15–24”, 1 = “25–34”, 2 = “35–44”, 3 = “45 and above”), Mother’s educational level (0 = “No education”, 1 = “Primary”, 2 = “Secondary”, 3 = “Higher”), Antenatal care visit (0 = “None”, 1 = “1–7”, 2 = “≥8”), Mother’s occupational status (0 = “No”, 1 = “Yes”), Marital Status of mother (0 = “Never in union”, 1 = “Married/Living With Partner”, 2 = “Separated”), Wealth index of Mothers (0 = “Poorest”, 1 = “Poorer”, 2 = “Middle”, 3 = “Richer”, 4 = “Richest”).

#### 2.3.5. Household-Level Factors

Source of drinking water (0 = “Improved Water”, 1 = “Un-Improved Water”), Type of toilet facility (0 = “Improved toilet/sanitation”, 1 = “Un-Improved toilet/Sanitation”), Type of Cooking fuel (0 = “Modern fuel”, 1 = “Traditional fuel”), Sex of household head (0 = “Male”, 1 = “Female”).

#### 2.3.6. Community-Level Factors

Place of residence (0 = “Urban”, 1 = “Rural”), Region (0 = “Tigray”, 1 = “Afar”, 2 = “Amhara”, 3 = “Oromia”, 4 = “Somali”, 5 = “Benishangul-gumuz”, 6 = “SNNPR”, 7 = “Gambela”, 8 = “Harari”, 9 = “Addis Ababa”, 10 = “Dire Dawa”).

### 2.4. Statistical Model

#### 2.4.1. Ordinal Logistic Regression

To identify the determining factors among the ordered response variable, the ordinal logistic regression was used and expressed as follows;

An alternative to least-squares regression that guarantees the fitted probabilities between 0 and 1 is the method of ordinal logistic regression. We arbitrarily designate the last group, group K, to serve as the baseline category. In the ordinal logit model;

$$f\left(Y_{j}\left(X\right)\right)=\log\left(\frac{f\left(y_{j}\left(X\right)\right)}{1-f\left(y_{j}\left(X\right)\right)}\right)=log\left(\frac{pr\left(Y\leq j\backslash X\right)}{pr(Y>j\backslash X)}\right)=\alpha_{j}+\beta X,j=1,2,\;\ldots ,\;K-1$$

$Y_{j}\left(X\right)=\frac{e^{\propto_{j}+\beta X}}{1+e^{\propto_{j}+\beta X}},$ Where j indexes cut-off points for categories (K) of the response variables, the function $f\left(Y_{j}\left(X\right)\right)$ is the link function that connects the systematic components of the linear model, $\propto_{j}$ represents a separate intercept for each cumulative probability and β represents the regression coefficient.

If multiple explanatory variables are applied to the ordinal regression model, βX is replaced by the linear combination of ($\propto_{j}+\beta_{1}X_{j1}+\beta_{2}X_{j2}+\cdots +\beta_{p}X_{jp}$).

##### Assumptions of the Ordinal Logistic Regression Model

Since the ordinal logistic regression model was fitted, the assumptions to ensure that it is a valid model were checked. The assumptions of the ordinal logistic regression are as follows and were tested in the following order:

1. The dependent variable is ordered.
2. One or more of the independent variables are either continuous, categorical or ordinal.
3. No multicollinearity.
4. Proportional odds.

##### The Proportional Odds Model (POM)

The proportional odds model assumes that the cumulative logits can be represented as parallel linear functions of independent variables. That is, for each cumulative logit, the parameters of the models should be the same, except for the intercept. Consequently, according to the proportional odds assumption, the odds ratio is the same for all categories of the response variable [26].

Given that the outcome categories of the dependent variable appear to be ordered in terms of the severity of anaemia, a typical approach is to use the standard ordered logit model called the proportional odds model.

The proportional odds model is the log odds of the first *k* −1 cumulative probabilities as:

$$logit\;[p\left(Y\leq i\right]=\;\log\left(\frac{p[\left(Y\leq i)\right]}{[1-p\left(Y\leq i)\right]}\right)=log\left[\frac{\pi_{ij}}{1-\pi_{ij}}\right]$$

And the relationship between the cumulative logits of Y is:

$$log\left[\frac{\pi_{ij}}{1-\pi_{ij}}\right]=log\left[\frac{\pi_{ij}}{\pi_{ij+1}+\cdots +\pi_{k}}\right];i=1,\;2,\;\ldots ,k-1$$

#### 2.4.2. Bivariate Analysis 

First, descriptive and bivariate analysis was performed. Bivariate analysis was used to select candidate variables with *p*-values less than 0.25 for multilevel ordinal regression models [27,28]. To analyze the bivariate analysis, the SPSS version 20 Statistical software and STATA version 12 were used for the multilevel analysis.

#### 2.4.3. Multilevel Ordinal Logistic Regression Model

##### Four-Level Multilevel Model

To identify the variations across the individual, household, maternal, and community level factors, the multilevel logistic regressions were applied as follows;

For this study, the authors used the community as level 4, the household as level 3, mothers as level 2, and individuals (child characteristics) as level 1 variables, respectively.

##### Multilevel Empty Ordinal Logistic Regression Model

This model only contains random groups and random variation within groups and between groups.

$$\eta_{ijkl}=\beta_{0}+\varepsilon_{jkl}+\varepsilon_{l}$$

Whereas, $\varepsilon_{jkl}$ denote the random effect for the $j^{th}$ level cluster, $k^{th}$ cluster in the $l^{th}$ level of cluster and $\varepsilon_{l}$ denote the random effect for the $l^{th}$ fourth-level cluster. 

For i = ,…, number of individuals, j = 1,…, number of mothers within each household in each community, k = 1,…, number of households in each community, and l = 1,…, number of community.

##### Random Intercept Multilevel Ordinal Logistic Regression Model

In this model, the covariates are included, and the intercept is the only random effect meaning that the groups differ with respect to the average value of the response variable.

$$\eta_{ijkl}=\beta_{0}+\beta_{1h}\overset{p}{\underset{h=1}{\sum }}X_{hijkl}+\beta_{2h}\overset{q}{\underset{h=1}{\sum }}X_{hjkl}+\beta_{3h}\overset{l}{\underset{h=1}{\sum }}X_{hkl}+\beta_{4h}\overset{m}{\underset{h=1}{\sum }}X_{hl}\varepsilon_{jkl}+\varepsilon_{jkl}+\varepsilon_{l}$$

Whereas, $X_{hijkl}$ denote the vector of the first level variables, $X_{hjkl}$ denote the vector of the second level variables, $X_{hkl}$ denote the vector of 3rd level predictor variables, and $X_{hl}$ denote the vector of 4th level predictor variables. In addition, $\beta_{1h}$ denote the vector of regression parameters for the first-level variables, $\beta_{2h}$ denote the vector of regression parameters for second-level variables, $\beta_{3h}$ denote the vector of regression parameters for the third-level variables, and $\beta_{4h}$ denote the vector of regression parameters for fourth-level variables. $\varepsilon_{jkl}$ denote the random effect for the j^th^ level cluster in the k^th^ level cluster in the $l^{th}$ level of cluster and $\varepsilon_{l}$ denote the random effect for the l^th^ 4^th^ level cluster.

For i = ,…, number of individuals, j = 1,…, number of mothers within each household in each community, k = 1,…, number of households in each community, and l = 1,…, number of community.

##### Random Coefficient Multilevel Ordinal Logistic Regression Model

In this model, the coefficients of the explanatory variables are considered as random.

$$\eta_{ijkl}=\beta_{0}+\beta_{1h}\overset{p}{\underset{h=1}{\sum }}X_{hijkl}+\beta_{2h}\overset{q}{\underset{h=1}{\sum }}X_{hjkl}+\beta_{3h}\overset{l}{\underset{h=1}{\sum }}X_{hkl}+\beta_{4h}\overset{m}{\underset{h=1}{\sum }}X_{hl}\varepsilon_{jkl}+\varepsilon_{jkl}+\varepsilon_{l}+\varepsilon_{ojl}\overset{p}{\underset{h=1}{\sum }}X_{hijkl}+\varepsilon_{l}+\varepsilon_{ol}\overset{p}{\underset{h=1}{\sum }}X_{hijkl}$$

Whereas, $X_{hijkl}$ denote the vector of the first level variables, $X_{hjkl}$ denote the vector of the second level variables, $X_{hkl}$ denote the vector of 3rd level predictor variables, and $X_{hl}$ denote the vector of 4th level predictor variables. In addition, $\beta_{1h}$ denote the vector of regression parameters for the first-level variables, $\beta_{2h}$ denote the vector of regression parameters for second-level variables, $\beta_{3h}$ denote the vector of regression parameters for the third-level variables, and $\beta_{4h}$ denote the vector of regression parameters for fourth-level variables. $\varepsilon_{ojl}$ and $\varepsilon_{ol}$ are the random slope. The part $\beta_{0}+\beta_{1h}\sum_{h=1}^{p}X_{hijkl}+\beta_{2h}\sum_{h=1}^{q}X_{hjkl}+\beta_{3h}\sum_{h=1}^{l}X_{hkl}+\beta_{4h}\sum_{h=1}^{m}X_{hl}\varepsilon_{jkl}$ are the fixed part of the model and $\varepsilon_{jkl}+\varepsilon_{l}+\varepsilon_{ojl}\sum_{h=1}^{p}X_{hijkl}+\varepsilon_{l}+\varepsilon_{ol}\sum_{h=1}^{p}X_{hijkl}$ are the random part of the model.

For i = ,…, number of individuals, j = 1,…, number of mothers within each household in each community, k = 1,…, number of households in each community, and l = 1,…, number of community.

### 2.5. Measures of Variation (Random Effects)

Intra Cluster Correlation

To understand the variation of childhood anaemia among the maternal, households, and communities, the researcher used intra-cluster correlation and determined as follows;

$$ICC_{maternal}=\frac{V_{maternal}}{V_{community}+V_{household}+V_{maternal}+\frac{\pi^{2}}{3}}$$

$$ICC_{household}=\frac{V_{household}}{V_{community}+V_{household}+V_{maternal}+\frac{\pi^{2}}{3}}$$

$$ICC_{community}=\frac{V_{community}}{V_{community}+V_{household}+V_{maternal}+\frac{\pi^{2}}{3}}$$

where $\;V_{community}$, $V_{household}$ and $V_{maternal}$ are the variances of childhood anaemia at the community, household, and maternal levels, respectively.

### 2.6. Proportional Change in Variance (PCV)

The proportional change in variance expresses the change in the area level variance between the intercept only model and the individual level model, and between the individual level model and the model further including the area level covariate and determined as follows;

$$PCV=\frac{{\hat{\tau }}_{null}-{\hat{\tau }}_{full}}{{\hat{\tau }}_{null}}$$

where ${\hat{\tau }}_{full}\;and\;{\hat{\tau }}_{null}$ denote the estimated variances of random-effects distributions for the full and null models, respectively.

### 2.7. Median Odds Ratio (MOR)

Measures of heterogeneity in logistic multilevel regression models can be determined as follows;

$$\mathrm{MOR}=exp\left(\sqrt{2{\hat{\tau }}^{2}}\times \Phi^{-1}\left(0.75\right)\right)$$

where ${\hat{\tau }}^{2}$ is the estimated variance of the distribution of the random effects, and Φ denotes the cumulative distribution function of the standard normal distribution, while $\Phi^{-1}$(0.75) = 0.6745 is the 75th percentile of a standard normal distribution.

### 2.8. Model Selection Criteria

#### Akaike Information Criterion (AIC)

Model suitability or fitting review is required prior to model fitting. Akaike Information Criterion (AIC) was used to select the best model. The model with the lowest AIC value is the best. The model with a small value of AIC is the optimal model, which means a model that is close to the actual one [23].

AIC is defined as:

AIC=−2ln(likelihood)+2k

where *k* is the degrees of freedom of the model, computed as the rank of the variance-covariance matrix of the parameters. N is the number of observations used in the estimation, or more precisely, the number of independent terms in the probability. That means an intercept-only/null model (Model I), which did not include any covariate but includes outcome variables, individual-level factors (Model II), mothers-level factors(Model III), household-level factors (Model IV), and community-level factors (Model V).

## 3. Results

The data used for this analysis was obtained from EDHS 2016 on the factors related to childhood anemia in Ethiopia. Descriptive statistics and multilevel ordinal logistic regression were applied to analyze the data.

Assumption Checking

1. The dependent variable or childhood anaemic status was ordered. i.e., mild, moderate, and severe.
2. One or more of the independent variables are either continuous, categorical or ordinal, as shown in the table below.
3. No multicollinearity: as shown in the table below, the multicollinearity among the individual, household, maternal, and community-level explanatory variables was tested using the variance Inflation Factor (VIF). Table 1 shows that the VIF for each of the explanatory variables was less than five (5). It shows the absence of multicollinearity in the models, i.e., indicating no multicollinearity problem in the data.
4. Conducting the Brant test of the parallel regression (proportional odds) assumption for the status of children’s anaemic status. We identified no predictors found to violate the proportional odds assumption (Table 1).

### 3.1. Descriptive Statistics on Factors Associated with Childhood Anaemia Status

From the overall sample of 7960 under-five children, 1950 (24.5%) had mild anaemia, 2264 (28.4%) had moderate anaemia, and 179 (2.2%) were severe anaemia without including the child’s normal anaemic status. Among the overall sample of 7960 under-five children, 984 (24.39%) were mild, 1146 (28.41%) were moderate, and 95 (2.3%) were severe, while 966 (24.6%) females were mild anaemia, 1118 (28.5%) females were moderate anaemia, and 84(2.1%) females were severe anaemia without including child normal anaemic status. Similarly, among children who were mild, moderate, and severe anaemia, 430 (30%), 456 (31.8%), and 41 (2.87%) were those who resided in urban areas, respectively, but 1520 (23.3%), 1808 (27.7%), and 138 (2.1%), children were those who resided in rural areas, respectively without including child normal anaemic status.

### 3.2. Inferential Statistical Analysis on Factors Associated with Childhood Anaemia Status

#### Bivariate Analysis

Bivariate analyses were performed on all independent variables separately from the outcome variables prior to multilevel analysis. The variables associated with *p*-values < 0.25 for anaemic status were then selected and entered into multilevel analyses. From the result in Table 2, the size of the child at birth, the mother’s marital status, and the type of cooking fuel variables are not candidates for multilevel analysis on childhood anaemic because of their *p*-value greater than 0.25.

### 3.3. Multilevel Ordinal Logistic Regression Analysis of Childhood Anaemic Status among Maternal, Household, and Community Factors

#### 3.3.1. Intercept Model Only

In this model, there is a parametric version of assessing heterogeneity of the childhood anaemic status among the maternal, household, and community factors.

From Table 3, the estimated variance for maternal, household, and community-level were $\sigma_{ou}{}^{2}$ = 1.546, $\sigma_{ou}{}^{2}=1.698\;$ and $\sigma_{ou}{}^{2}=1.425$, respectively, which was significantly different from zero, indicating variations of childhood anaemic across maternal, household, and community levels of Ethiopia. The result of ICC is 0.194, 0.213, and 0.179 for maternal, household, and community, respectively. This suggests that about 19.4%, 21.3%, and 17.9% of the variation in childhood anaemic were due to the variation across the maternal, household, and community level factors, respectively.

#### 3.3.2. Model Comparison

Once the set of candidate models has been chosen, the statistical analysis allows us to select the best of these models. Good model selection is a balance between simplicity and goodness of fit. Choosing are levant multilevel model is therefore an important step and should be based on the need for model parsimony.

The smallest value of AIC indicates a better model. From the result of Table 4, the AIC for multilevel ordinal logistic regression of model VI is 12,368.45, which is small compared to the rest model. Therefore, it better fits the data to predict childhood anaemia status in Ethiopia. This suggests that the multilevel logistic regression of model VI with the fixed explanatory variables and random effects is a better model than the other. Thus, the interpretations of the parameter and conclusion of the finding were based on model VI.

### 3.4. Multilevel Ordinal Logistic Regression Model Result on Childhood Anaemia Status

It is possible to generalize the model so that the effect of the level one covariate is different in each mother, household, and community. This approach can be made by adding random coefficients in front of some of the individual-level covariates of the model. The following table presents some random coefficients and fixed explanatory variables with a significant effect on childhood anaemic in Ethiopia.

The child’s age (in months), sex of the child, preceding birth interval (in months), mother’s educational level, antenatal care visit, wealth index of mothers, source of drinking water, type of toilet facility, place of residence, and region were significant predictors of the childhood anaemic status at a 5% level of significance.

The odds of the status of child anaemia exposure to severe status were less likely in females(OR: 0.63) than males, and the odds of child anaemic status exposed to mild and moderate status were more likely in females(OR: 1.61), in comparison with the reference category relative to other anaemic status controlling for other variables in the model. The odds of child anaemic exposed to mild status were less likely in a child with age between 38 and 47 and between 48 and 59 months (OR: 0.81, OR: 0.79), respectively, in comparison with the reference category relative to the other anaemic status; The odds of child anaemic exposed to moderate status were less likely in a child with age between 38 and 47 and between 48 and 59 months (OR: 0.80, OR: 0.78), respectively, in comparison with the reference category relative to the other anaemic status; The odds of child anaemic exposed to sever status were less likely in a child with age between 38 and 47 and between 48 and 57 months (OR: 0.79, OR: 0.87), respectively, in comparison with the reference category relative to other anaemic status controlling for other variables in the model. The odds of child anaemic exposure to mild, moderate, and severe were more likely in mothers with primary education level (OR: 1.4, OR: 1.56, OR: 1.01), respectively, in comparison with the reference category relative to each other controlling for other variables in the model. The odds of child anaemic exposure to mild, moderate, and severe were less likely in 1–7 antenatal care visits(OR: 0.08, OR: 0.021, OR: 0.05), respectively, in comparison with the reference category relative to each other controlling for other variables in the model. The odds of child anaemic exposure to mild, moderate, and severe were more likely in the richest wealth index of the mother (OR: 1.44, OR: 1.36, OR: 1.4), respectively, in comparison with the reference category relative to each other controlling for other variables in the model. The odds of child anaemic exposure to mild, moderate, and severe were less likely in the un-improved water source of drinking water (OR: 0.57, OR: 0.49, OR: 0.62), respectively, in comparison with the reference category relative to each other controlling for other variables in the model. The odds of child anaemic exposure to mild, moderate, and severe were less likely in the un-improved toilet facility (OR: 0.66, OR: 0.64, OR: 0.65), respectively, in comparison with the reference category relative to each other controlling for other variables in the model. The odds of child anaemic exposure to mild, moderate, and severe were less likely in the rural residence (OR: 0.70, OR: 0.81, OR: 0.56), respectively, in comparison with the reference category relative to each other controlling for other variables in the model. The odds of child anaemic exposure to mild, moderate, and severe were less likely in the Affar region (OR: 0.68, OR: 0.72, OR: 0.0.70), Amhara region (OR: 0.57, OR: 0.42, OR: 0.56), respectively, and were more likely in the SNNPR (OR:1.12, OR:1.14, OR:1.15) and Addis Ababa (OR: 3.65, OR: 2.67, OR: 2.89), respectively, in comparison with the reference category relative to each other controlling for other variables in the model.

In the final model, the variation between women’s, household, and community on childhood anaemia concluded that there is a maternal variation of 15.06%, household variation of 15.6%, and community variation of 14.22% and close to half of the variance across the communities was explained by the child-, maternal-, household-, and community-level factors (PCV = 40.42%). Importantly, as determined by a proportional change in variance, 59.58% of the community-level variance of childhood anaemia was accounted for by the joint effects of maternal, household, and community-level factors.

Table 5 reveals that the addition of individual, maternal level, household level, and community level variables to the model at the same time leads to decreases in the ICC at all levels. This shows that about 15.06%, 15.6% and 14.2% variation in the prevalence of childhood anaemia is due to the difference in the maternal level, household level and community level, respectively.

## 4. Discussion

The study examined the inequalities and factors associated with childhood anaemia using a nationally representative EDHS dataset. We found that the prevalence of mild, moderate, and severe childhood anaemia was24.5%, 28.4%, and 2.2%, respectively.

The sex of the child was found to be significantly associated with anaemia. Specifically, females were more likely to be anaemic compared to their male counterparts in mild and moderate anaemia, but an inverse association was found regarding severe anaemia. Contrary to our findings, Venda [29] reported that male children were more likely to be anaemic. Previous studies conducted in Ethiopia to examine childhood anaemia found consistent findings [30,31]. The inconsistencies could be due to the sociocultural norms and differentials regarding the intake of iron-rich foods by gender and age. Also, household food security and other childhood infections could have played a key role in the observed association found in the study. Further studies are recommended to investigate the intrinsic association between the sex of a child and their anaemic status.

Further, the child’s age (in months) was a significant factor in childhood anaemia. A similar study conducted by Booth & Aukett and Lanzkowsky suggests that children aged 6–11 months had significantly higher odds of being anaemic. The study states that by 4 months of age, neonatal iron stores are usually reduced by half [5,32], and by 6 months, children have depleted the iron stores present at birth [4,33].

Maternal educational level has a significant effect on childhood anaemic in this study. The study conducted in Ethiopia by [19,34] confirmed this study and suggested that mothers are mostly caregivers for their children and that maternal education has always been linked to many child health outcomes. It may also affect health decision-making and thus influence the probability of a child meeting certain nutrition-related requirements [4,5]. In addition, in developing countries, there is a high prevalence of iron deficiency anaemia, which reduces cognitive performance, work performance, and endurance; it also causes learning difficulties and has a negative impact on the development of the infant population.

The wealth index is also the other significant factor in childhood anaemia in this study. Similar findings conducted in India and Maryland [35,36] suggest that the household wealth index directly influences broader socioeconomic conditions directly on hemoglobin levels among children and hence childhood anaemia. This condition has been attributed to generalized bone marrow failure resulting from malnutrition, other micronutrient deficiencies, contact with biofuel smoke, and mechanisms linked to lower-income and social statuses [6]. A similar finding from another study conducted in Ethiopia confirmed this study and suggested that households with a higher wealth quintile are more likely to provide balanced macronutrients and micronutrients to their children. Children from these households have more chances of accessing health care services [37]. Several studies confirm that children from a lower economic status are vulnerable to various nutritional disorders, including anaemia, and are at risk of easily preventable diseases [13].

The type of toilet facility has a significant effect on childhood anaemia in this study. Similar findings conducted in Lesotho [38] suggest that a better understanding of fuel usage in households can undeniably lead to the development of interventions and policies that can reinforce proper fuel usage and significantly reduce the prevalence of anaemia.

### Limitations

The current study findings should be interpreted with caution. The cross-sectional nature of the data restricts causality from the observed findings. Moreover, the DHS data lacks detailed information on the other risk factors of anaemia, such as malaria, intestinal parasite, and dietary intake markers such as macro and micronutrients connected to anemia. We only used Hb concentration (in g/L) to determine childhood anaemia status; hence, we could not ascertain whether the anemia was caused by other factors such as iron deficiency.

## 5. Conclusions

The study has shown that anaemia is prevalent among children in Ethiopia with variation across maternal, household, and community characteristics. The result revealed that anaemia among the children was significantly associated with the age of the child, sex of the child, birth interval, the mother’s educational level, antenatal care attendance, wealth index, source of drinking water, type of toilet facility, place of residence, and region.Therefore, appropriate and tailored interventions are required to reduce the prevalence of childhood anaemia. These interventions include improving women’s access to education, providing health education on child feeding practices (e.g., complimentary feeding), and strengthening social support systems (e.g., free access to maternal healthcare). In light of the identified factors of childhood anaemia, a pragmatic approach is required from policymakers. Further research is needed to understand the risk factors and aetiologies of anaemia across different settings in Ethiopia.

## Figures and Tables

**Table 1:** Proportional odds assumption checking.

**Co-Variable**	**Regression Coefficient**	VIF	Brant Test (*p*-Value)	95% Confidence Interval	
				Lower Bound	Upper Bound
[Mothers occupational status = No]	−0.089	1.063	0.090	−0.191	0.014
[Mothers occupational status = Yes]	0 ^a^				
[Sex of child = Male]	0.010	2.665	0.814	−0.076	0.097
[Sex of child = Female]	0 ^a^				
[Current marital status = Never in Union]	−0.354	1.571	0.584	−1.622	0.913
[Current marital status = Married/living with partner]	−0.253		0.203	−0.643	0.137
[Current marital status = separated]	0 ^a^				
[Age of Mothers = 15–24]	−5.484	1.069	0.300	−6.213	−4.754
[Age of Mothers = 25–34]	−2.301		0.089	−3.036	−1.566
[Age of Mothers = 35–44]	−4.038		0.063	−4.764	−3.312
[Age of Mothers = 45 and above]	0 ^a^				
[Wealth index of mothers = Poorest]	−0.225	1.923	0.078	−0.437	−0.013
[Wealth index of mothers = Poorer]	−0.186		0.085	−0.399	0.026
[Wealth index of mothers = Middle]	−0.252		0.091	−0.467	−0.037
[Wealth index of mothers = Richer ]	−0.110		0.879	−0.322	0.102
[Wealth index of mothers = Richest ]	0 ^a^				
[Child Age (in Months) = <6]	0.003	1.027	0.967	−0.157	0.163
[Child Age (in Months = 6–11]	−0.016		0.853	−0.181	0.150
[Child Age (in Months = 12–23]	−0.035		0.632	−0.177	0.107
[Child Age (in Months = 24–37]	0.020		0.775	−0.116	0.156
[Child Age (in Months = 38–47]	0.055		0.773	−0.096	0.206
[Child Age (in Months = 48–59]	0 ^a^				
[Region = Tigray]	−0.101	1.186	0.921	−0.348	0.146
[Region = Afar]	0.099		0.749	−0.157	
[Region = Amhara]	−0.267		0.941	−0.523	−0.011
[Region = Oromia]	−0.397		0.801	−0.640	−0.154
[Region = Somalia]	0.416		0.061	0.178	0.654
[Region = Benishangul-gumuz]	−0.323		0.717	−0.588	−0.058
[Region = SNNPR]	−0.034		0.787	−0.280	0.212
[Region = Gambela]	−0.306		0.630	−0.582	−0.030
[Region = Harari]	0.132		0.650	−0.145	0.409
[Region = Addis Ababa]	−0.079		0.627	−0.395	0.238
[Region = Dire dawa]	0 ^a^				
[Place of residence = Urban]	−0.239	3.140	0.816	−0.433	−0.045
[Place of residence = Rural]	0 ^a^				
[Mothers Educational level = No education]	0.176	1.875	0.654	−0.126	0.477
[Mothers Educational level = primary]	0.246		0.902	−0.049	0.541
[Mothers Educational level = secondary]	0.124		0.618	−0.176	0.425
[Mothers Educational level = higher]	0 ^a^				
[Sex of household head = Male]	−0.012	1.134	0.845	−0.130	0.107
[Sex of household head = Female]	0 ^a^				
[Size of child at birth = Very large]	−0.221	1.045	0.615	−0.373	−0.068
[Size of child at birth = larger than Average]	−0.088		0.778	−0.247	0.071
[Size of child at birth = Medium]	−0.072		0.663	−0.199	0.054
[Size of child at birth = Smaller than average]	−0.048		0.691	−0.222	0.126
[Size of child at birth = Very Small]	0 ^a^				
[Preceding birth interval (months) = <24]	−0.043	1.029	0.803	−0.145	0.058
[Preceding birth interval (months = ≥24]	0 ^a^				
[Antenatal care visit = None]	0.604	1.464	0.501	0.243	0.964
[Antenatal care visit = 1–7]	0.254		0.558	−0.055	0.563
[Antenatal care visit = ≥8]	0 ^a^				
[Type of cooking fuels = Modern fuel]	−0.085	1.350	0.664	−0.314	0.143
[Type of cooking fuels = Traditional fuel]	0 ^a^				
[Type of toilet facility = Improved toilet facility]	−0.034	1.522	0.656	−0.184	0.116
[Type of toilet facility = Un-improved toilet facility]	0 ^a^				
[Source of drinking water = Improved water]	0.097	1.201	0.056	−0.002	0.197
[Source of drinking water = Un-improved water]	0 ^a^				

^a^ = reference category. Overall Brant test of parallel regression assumption: Chi-square = 48.66, *p*-value = 0.025. Goodness-of-fit test of overall model (Likelihood Ratio): Chi-square = 756.21, *p*-value = 0.000.

**Table 2:** Summaries of the descriptive statistics and bivariate analysis on factors associated with childhood anaemia status.

Variables	Categories	Anaemic Status			
		Mild	Moderate	Severe	$x^{2}$*p*-Value
Place of residence	Urban	430	456	41	*p* < 0.000 *
	Rural	1520	1808	138	
Mothers Educational level	No education	1209	1466	93	*p* < 0.000 *
	Primary	497	552	59	
	Secondary	160	146	20	
	Higher	84	100	7	
Sex of household head	Male	1542	1771	141	*p* < 0.000 *
	Female	408	493	38	
Wealth index of mothers	Poorest	660	835	62	*p* < 0.000 *
	Poorer	363	342	34	*p* < 0.000 *
	Middle	253	299	21	
	Richer	226	306	10	
	Richest	448	482	52	
Age of Mothers	15–24	10	1023	83	*p* < 0.000 *
	25–34	1939	867	68	
	35–44	1	367	8	
	45 and above	0	7	20	
Current marital status	Never in Union	3	2	1	
	Married/living with partner	1871	2186	176	0.987
	Separated	76	76	2	
Region	Tigray	215	226	24	*p* < 0.000 *
	Afar	147	250	5	
	Amhara	179	199	5	
	Oromia	298	267	13	
	Somalia				
	Benishangul-gumuz	279	359	59	
	SNNPR	132	170	8	
	Gambela	261	287	31	
	Harari	135	125	1	
	Addis Ababa	121	135	7	
	Dire dawa	92	133	23	
Mothers occupational status	No	1425	1617	128	*p* < 0.000 *
	Yes	525	647	51	
Sex of child	Male	984	1146	95	*p* < 0.000 *
	Female	966	1118	84	
Preceding birth interval (months)	<24	535	593	48	*p* < 0.000 *
	≥24	1415	1671	131	
Size of the child at birth	Very large	321	364	19	0.581
	larger than Average	288	310	33	
	Medium	829	968	82	
	Smaller than average	185	216	13	
	Very Small	327	406	32	
Child Age (in Months)	<6	271	284	22	*p* < 0.000 *
	6–11	235	255	18	
	12–23	373	442	31	
	24–37	465	512	54	
	38–47	275	362	28	
	48–59	331	409	26	
Antenatal care visit	None	116	89	40	*p* < 0.000 *
	1–7	1728	2063	131	
	≥8	106	112	8	
Source of drinking water	Improved water	1203	1450	116	*p* < 0.000 *
	Un-improved water	116	814	63	
Type of toilet facility	Improved toilet facility	363	426	53	*p* < 0.000 *
	Un-improved toilet facility	1587	1838	126	
Type of cooking fuels	Modern fuel	147	159	20	0.615
	Traditional fuel	1803	2105	159	

* Chi-square was significant at (*p* < 0.25).

**Table 3:** Results of multilevel logistic regression of intercept-only model.

Model	Coefficient	Standard Error	Z-Value	*p*-Value
Fixed intercept (β_0_)	−0.781	0.099	−7.88	0.000
Random effect				
Variance (community)	1.425	0.213		
Variance (household)	1.698	0.254		
Variance (maternal)	1.546	0.651		
Icc (community)	0.179			
Icc (household)	0.213			
Icc (maternal)	0.194			

**Table 4:** Model comparisons among multilevel multinomial models.

Model Comparison Criteria	Null Model (Model I)	Individual-Level Factors (Model II)	Maternal Level Factors (Model III)	Households Level Factors (Model IV)	Community-Level Factors (Model V)	Individual, Maternal, Household, and Community-Level Factors (Model VI)
AIC	19,059.34	21,025.69	14,191.21	15,124.87	15,001.54	12,368.45

AIC: Akaike Information Criteria.

**Table 5:** Results of multilevel logistic regression model on childhood anaemia status.

Variable	Mild		Moderate		Severe	
Fixed Part	OR[95% C.I]	*p*-Value	OR[95% C.I]	*p*-Value	OR[95% C.I]	*p*-Value
Constant	2.8[2.0–3.9]	0.000	2.58[2.24–2.96]	0.000	3.09[2.03–4.70]	0.000
Individual-level factors						
Child Age (in Months) (ref. < 6)						
6–11	0.96[0.73–1.10]	0.329	0.91[0.75–1.11]	0.397	0.91[0.75–1.12]	0.406
12–23	0.86[0.72–1.03]	0.120	0.87[0.73–1.04]	0.130	0.82[0.72–1.03]	0.115
24–37	0.83[0.70–0.98]	0.035	0.86[0.70–0.99]	0.000	0.84[0.71–1.0]	0.057
38–47	0.81[0.67–0.97]	0.029	0.80[0.65–0.94]	0.000	0.79[0.66–0.94]	0.011
48–59	0.79[0.66–0.95]	0.012	0.78[0.65–0.93]	0.000	0.87[0.71–0.98]	0.000
Sex of child (ref. Male)						
Female	1.54[1.04–1.99]	0.000	1.61[1.24–1.89]	0.000	0.63[0.31–0.99]	0.000
Preceding birth interval (months) (ref. < 24)						
≥24	0.51[0.33–0.87]	0.004	0.89[0.80–0.99]	0.048	0.40[0.14–0.81]	0.001
Maternal level factors						
Mother’s age (ref. 15–24)						
25–34	1.1[0.97–1.31]	0.093	1.01[0.95–1.19]	0.229	0.73[0.58–1.79]	0.681
35–44	1.06[0.91–1.25]	0.406	0.64[0.40–1.68]	0.129	0.41[0.29–1.44]	0.149
45 and above	1.14[0.97–1.35]	0.101	0.51[0.42–1.58]	0.409	0.26[0.15–1.05]	0.331
Mother’s educational level (ref. No education)						
Primary	1.4[0.86–1.99]	0.000	1.56[0.74–1.48]	0.547	1.01[0.91–1.1]	0.628
Secondary	1.27[0.97–1.34]	0.000	1.27[0.56–1.74]	0.241	1.8[1.45–2.3]	0.000
Higher	1.2[0.73–1.48]	0.000	1.32[1.09–1.9]	0.000	3.3[2.22–4.96]	0.000
Antenatal care visit (ref. None)						
1–7	0.03[0.008–0.14]	0.000	0.06[0.009–0.15]	0.000	0.01[0.009–0.20]	0.000
≥8	0.08[0.019–0.16]	0.000	0.021[0.01–0.12]	0.000	0.05[0.010–0.14]	0.000
Mother’s occupational status (ref. No)						
Yes	0.98[0.88–1.10]	0.781	0.92[0.83–1.03]	0.191	0.96[0.86–1.07]	0.495
Wealth index (ref. poorest)						
Poorer	1.01[0.90–1.1]	0.763	1.01[0.88–1.17]	0.778	1.02[0.89–1.1]	0.718
Middle	1.78[1.40–2.2]	0.000	0.94[0.81–1.09]	0.462	0.95[0.82–1.103]	0.535
Richer	3.11[2.0–4.6]	0.000	0.98[0.84–1.14]	0.829	1.01[0.86–1.1]	0.870
Richest	1.44[1.15–2.1]	0.000	1.36[1.15–1.59]	0.000	1.4[1.11–1.75]	0.003
Household-level factors						
Sex of household head (ref. Male)						
Female	0.53[0.28–1.34]	0.065	0.65[0.41–1.42]	0.334	0.25[0.18–1.39]	0.091
Source of drinking water (ref. Improved Water)						
Unimproved Water	0.57[0.18–0.88]	0.001	0.49[0.29–0.79]	0.038	0.62[0.43–0.91]	0.022
Type of toilet facility (ref. Improved toilet)						
Unimproved toilet	0.66[0.57–0.78]	0.000	0.64[0.55–0.75]	0.000	0.65[0.55–0.76]	0.000
Community level factors						
Place of residence (ref. urban)						
Rural	0.70[0.60–0.82]	0.000	0.81[0.65–0.99]	0.000	0.56[0.48–0.64]	0.000
Region (ref. Tigray)						
Afar	0.68[0.55–0.85]	0.001	0.72[0.57–0.89]	0.003	0.70[0.56–0.87]	0.001
Amhara	0.57[0.46–0.71]	0.000	0.42[0.19–0.82]	0.000	0.56[0.41–0.75]	0.000
Oromia	0.45[0.37–0.55]	0.000	0.47[0.39–0.57]	0.000	0.47[0.39–0.57]	0.000
Somali	0.98[0.79–1.20]	0.860	1.01[0.82–1.25]	0.854	0.96[0.78–1.19]	0.772
Benishangul-gumuz	0.70[0.56–0.88]	0.003	0.72[0.58–0.91]	0.006	0.72[0.57–0.91]	0.005
SNNPR	1.12[0.91–1.39]	0.268	1.14[0.92–1.41]	0.218	1.15[0.93–1.42]	0.194
Gambela	0.84[0.66–1.07]	0.174	0.76[0.59–0.97]	0.000	0.81[0.64–1.04]	0.114
Harari	0.87[0.67–1.14]	0.338	0.83[0.64–1.09]	0.190	0.84[0.64–1.09]	0.201
Addis Ababa	3.65[2.4–5.4]	0.000	2.67[1.75–4.05]	0.000	2.89[1.8–4.44]	0.000
Diredawa	0.56[0.43–0.72]	0.000	0.54[0.42–0.71]	0.000	0.53[0.39–0.67]	0.000
Random-effect						
Var (Cons.) Community	0.849					
ICC for Community (%)	14.22%		PCV for Community (%)		40.42%	
Var (Cons.) Household	0.931					
ICC for Household(%)	15.6%		PCV for Household (%)		45.17%	
Var (Cons.) Mothers	0.899					
ICC for Mothers(%)	15.06%		PCV for Maternal (%)		41.85%	

## Data Availability

The dataset used is freely available to download at: https://dhsprogram.com/data/dataset/Ethiopia_Standard-DHS_2016.cfm?flag=1 (accessed on 8 January 2022).
